# (11a*S*)-7-Bromo-2,3,5,10,11,11a-hexa­hydro-1*H*-pyrrolo­[2,1-*c*][1,4]benzodiazepine-3,11-dione

**DOI:** 10.1107/S1600536811052962

**Published:** 2011-12-14

**Authors:** Chao Ma, Zhen-Zhong Wang, Li Pan, Yu Tian, Wei Xiao

**Affiliations:** a School of Pharmaceutical Engineering, Shenyang Pharmaceutical University, Shenyang 110016, People’s Republic of China; b Jiangsu Kanion Pharmaceutical Co. Ltd, Lianyungang 222001, People’s Republic of China

## Abstract

The title compound, C_12_H_11_BrN_2_O_2_, was prepared by an intra-cyclization reaction of (*S*)-1-(5-bromo-2-nitro­benz­yl)-5-oxopyrrolidine-2-carb­oxy­lic acid methyl ester in the presence of EtOH/Fe. The five-membered pyrrolidinone ring adopts an approximate envelope conformation, while the seven-membered diazepanone ring displays a twisted boat conformation. Inter­molecular classical N—H⋯O hydrogen bonds and weak C—H⋯O inter­actions help to stabilize the crystal structure.

## Related literature

For applications of pyrrolo­[2,1-*c*][1,4]benzodiazepines, see: Bose *et al.* (1992 ▶); Hu *et al.* (2001 ▶); Jitendra *et al.* (2007 ▶); Kamal *et al.* (2002 ▶); Thurston & Bose (1994 ▶). For a related structure, see: Cheng *et al.* (2007 ▶).
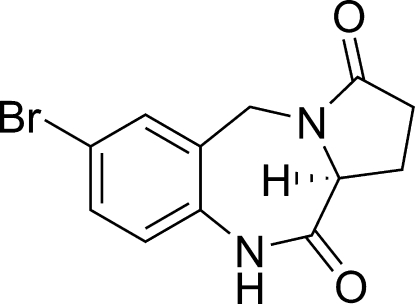


## Experimental

### 

#### Crystal data

C_12_H_11_BrN_2_O_2_
*M* *_r_* = 295.14
Orthorhombic, 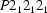

*a* = 4.3880 (4) Å
*b* = 13.1210 (11) Å
*c* = 19.8722 (16) Å
*V* = 1144.14 (17) Å^3^
*Z* = 4
Mo *K*α radiation
μ = 3.58 mm^−1^
*T* = 185 K
0.22 × 0.18 × 0.07 mm

#### Data collection

Bruker APEX CCD area-detector diffractometer
Absorption correction: multi-scan (*SADABS*; Bruker, 2001 ▶) *T*_min_ = 0.506, *T*_max_ = 0.788
7012 measured reflections
2244 independent reflections
2074 reflections with *I* > 2σ(*I*)
*R*_int_ = 0.021

#### Refinement

*R*[*F* ^2^ > 2σ(*F* ^2^)] = 0.022
*wR*(*F* ^2^) = 0.057
*S* = 1.02
2244 reflections
154 parameters
H-atom parameters constrained
Δρ_max_ = 0.49 e Å^−3^
Δρ_min_ = −0.21 e Å^−3^
Absolute structure: Flack (1983 ▶), 884 Friedel pairs
Flack parameter: −0.007 (9)

### 

Data collection: *SMART* (Bruker, 2007 ▶); cell refinement: *SAINT* (Bruker, 2007 ▶); data reduction: *SAINT*; program(s) used to solve structure: *SHELXTL* (Sheldrick, 2008 ▶); program(s) used to refine structure: *SHELXTL*; molecular graphics: *SHELXTL*; software used to prepare material for publication: *SHELXTL*.

## Supplementary Material

Crystal structure: contains datablock(s) I, global. DOI: 10.1107/S1600536811052962/xu5404sup1.cif

Structure factors: contains datablock(s) I. DOI: 10.1107/S1600536811052962/xu5404Isup2.hkl

Supplementary material file. DOI: 10.1107/S1600536811052962/xu5404Isup3.cml

Additional supplementary materials:  crystallographic information; 3D view; checkCIF report

## Figures and Tables

**Table 1:** Hydrogen-bond geometry (Å, °)

*D*—H⋯*A*	*D*—H	H⋯*A*	*D*⋯*A*	*D*—H⋯*A*
N2—H2⋯O1^i^	0.88	2.00	2.864 (2)	169
C5—H5*A*⋯O2^ii^	0.99	2.38	3.328 (3)	160

Symmetry codes: (i) 
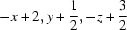
; (ii) 
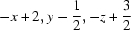
.

**Fig. 1.**
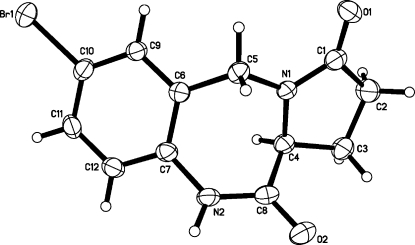
The structure of the title compound, showing 30% probability displacement ellipsoids and the atom-numbering scheme.

**Fig. 2.**
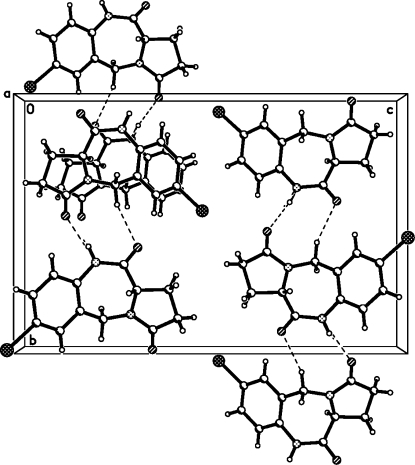
The molecular packing of the title compound.

## supplementary crystallographic information

### Comment

Pyrrolo[2,1-*c*][1,4]benzodiazepines (PBDs) are of considerable interest because of their wide range of biological activities such as antitumor agents, gene regulators, DNA probes, and anti-ischemic agents (Bose *et al.*, 1992; Hu *et al.*, 2001; Jitendra *et al.*,2007; Kamal *et al.*, 2002; Thurston & Bose, 1994). As PBDs compounds are of great pharmaceutical importance, we determined the title chiral compound's crystal structure. The molecular is shown in Fig. 1 and the bond lengths and angles are within normal ranges. PBD ring involes in a twisted conformation, similar to a related structure (Cheng *et al.*, 2007). The seven-membered ring C5—C6—C7—N2—C8—C4—N1 (substituted diazepine) is far from planar, and its shape approximates to a twist boat. In this description applied to the title compound (Fig. 1), atoms C5, C8, N1 and N2 form the bottom of the boat (deviation from the mean N1/C5/N2/C8 plane = 0.138 (5) Å), C4 the prow, and C6 and C7 the stern [deviations from the C5/C8/N1/N2 mean plane = 0.641, 0.854, 0.952 Å, respectively]. The bond length of the carbonyl groups C8=O2 and C1=O1 of 1.221 (2) and 1.233 (3) Å, respectively, are somewhat longer than typical carbonyl bonds. This may be due to the fact that atoms O1 and O2 participate in intermolecular van der Waals forces. The five-membered ring N1—C1—C2—C3—C4 (substituted pyrrole) is non-planar and adopts nearly envelope conformation (deviation from the mean C4/N1/C1/C2 plane = 0.019 Å). The C3 atom is located above the plane [deviations from the C4/N1/C1/C2 mean plane = 0.322 Å]. Atom C4 of the title molecule is chiral: S configuration was assigned to this atom based on the known chirality of the equivalent atom in the starting material. In the crystal structure, intermolecular C—H···O and N—H···O hydrogen bonds link the molecules together (Table 1) and help to stabilize the structure.

### Experimental

(*S*)-1-(5-bromo-2-nitrobenzyl)-5-oxopyrrolidine-2-carboxylic acid methyl ester (10.68 g, 30 mmol) was dissolved in ethanol (200 ml). Fe (3.92 g, 70 mmol) was added and the solution was heated to reflux for 50 min. The mixture was filtered and the filtrate was concentrated under vacuum. The pure product was obtained through silica gel chromatography (eluant: petroleum ether/ethyl acetate, 2:1). Single crystals suitable for X-ray diffraction were obtained by slow evaporation of a dilute solution of the title compound in ethyl acetate at room temperature.

### Refinement

All H atoms were placed in geometrically idealized positions with N—H = 0.88 Å and C—H = 0.95–1.00 Å, and refined in riding mode with *U*_iso_(H) = 1.2U_eq_(C,N).

### Crystal data

**Table:**

C_12_H_11_BrN_2_O_2_	*F*(000) = 592
*M**_r_* = 295.14	*D*_x_ = 1.713 Mg m^−^^3^
Orthorhombic, *P*2_1_2_1_2_1_	Mo *K*α radiation, λ = 0.71073 Å
Hall symbol: P 2ac 2ab	Cell parameters from 3967 reflections
*a* = 4.3880 (4) Å	θ = 2.6–26.0°
*b* = 13.1210 (11) Å	µ = 3.58 mm^−^^1^
*c* = 19.8722 (16) Å	*T* = 185 K
*V* = 1144.14 (17) Å^3^	Plate, colorless
*Z* = 4	0.22 × 0.18 × 0.07 mm

### Data collection

**Table:**

Bruker APEX CCD area-detector diffractometer	2244 independent reflections
Radiation source: fine-focus sealed tube	2074 reflections with *I* > 2σ(*I*)
graphite	*R*_int_ = 0.021
φ and ω scans	θ_max_ = 26.0°, θ_min_ = 1.9°
Absorption correction: multi-scan (*SADABS*; Bruker, 2001)	*h* = −5→5
*T*_min_ = 0.506, *T*_max_ = 0.788	*k* = −14→16
7012 measured reflections	*l* = −23→24

### Refinement

**Table:**

Refinement on *F*^2^	Secondary atom site location: difference Fourier map
Least-squares matrix: full	Hydrogen site location: inferred from neighbouring sites
*R*[*F*^2^ > 2σ(*F*^2^)] = 0.022	H-atom parameters constrained
*wR*(*F*^2^) = 0.057	*w* = 1/[σ^2^(*F*_o_^2^) + (0.0236*P*)^2^] where *P* = (*F*_o_^2^ + 2*F*_c_^2^)/3
*S* = 1.02	(Δ/σ)_max_ = 0.001
2244 reflections	Δρ_max_ = 0.49 e Å^−^^3^
154 parameters	Δρ_min_ = −0.21 e Å^−^^3^
0 restraints	Absolute structure: Flack (1983), 884 Friedel pairs
Primary atom site location: structure-invariant direct methods	Flack parameter: −0.007 (9)

### Special details

**Table:**

Geometry. All e.s.d.'s (except the e.s.d. in the dihedral angle between two l.s. planes) are estimated using the full covariance matrix. The cell e.s.d.'s are taken into account individually in the estimation of e.s.d.'s in distances, angles and torsion angles; correlations between e.s.d.'s in cell parameters are only used when they are defined by crystal symmetry. An approximate (isotropic) treatment of cell e.s.d.'s is used for estimating e.s.d.'s involving l.s. planes.
Refinement. Refinement of *F*^2^ against ALL reflections. The weighted *R*-factor *wR* and goodness of fit *S* are based on *F*^2^, conventional *R*-factors *R* are based on *F*, with *F* set to zero for negative *F*^2^. The threshold expression of *F*^2^ > σ(*F*^2^) is used only for calculating *R*-factors(gt) *etc*. and is not relevant to the choice of reflections for refinement. *R*-factors based on *F*^2^ are statistically about twice as large as those based on *F*, and *R*- factors based on ALL data will be even larger.

### Fractional atomic coordinates and isotropic or equivalent isotropic displacement parameters (Å^2^)

**Table:**

	*x*	*y*	*z*	*U*_iso_*/*U*_eq_	
Br1	0.25401 (6)	0.050666 (16)	0.521650 (10)	0.04269 (9)	
O1	1.0495 (4)	0.02986 (12)	0.85625 (8)	0.0421 (4)	
O2	0.9421 (4)	0.41192 (12)	0.82097 (8)	0.0443 (4)	
N1	0.8206 (4)	0.16602 (12)	0.80547 (8)	0.0238 (4)	
N2	0.7499 (5)	0.35871 (11)	0.72148 (7)	0.0275 (3)	
H2	0.8076	0.4163	0.7027	0.033*	
C1	0.8919 (5)	0.10757 (16)	0.85843 (11)	0.0301 (5)	
C2	0.7399 (7)	0.15233 (16)	0.92027 (10)	0.0388 (5)	
H2A	0.8826	0.1537	0.9588	0.047*	
H2B	0.5574	0.1123	0.9329	0.047*	
C3	0.6517 (5)	0.26009 (17)	0.89928 (10)	0.0322 (5)	
H3A	0.8033	0.3101	0.9157	0.039*	
H3B	0.4489	0.2785	0.9175	0.039*	
C4	0.6457 (4)	0.25772 (15)	0.82215 (10)	0.0253 (5)	
H4	0.4310	0.2502	0.8060	0.030*	
C5	0.9226 (5)	0.14577 (17)	0.73654 (10)	0.0249 (4)	
H5A	0.9572	0.0717	0.7312	0.030*	
H5B	1.1195	0.1808	0.7289	0.030*	
C6	0.6986 (4)	0.18057 (14)	0.68438 (9)	0.0214 (4)	
C7	0.6198 (5)	0.28407 (15)	0.67832 (10)	0.0240 (4)	
C8	0.7940 (5)	0.35007 (14)	0.78876 (10)	0.0276 (4)	
C9	0.5797 (5)	0.11109 (15)	0.63852 (10)	0.0233 (4)	
H9	0.6271	0.0407	0.6424	0.028*	
C10	0.3923 (5)	0.14489 (17)	0.58721 (10)	0.0267 (4)	
C11	0.3113 (5)	0.24598 (17)	0.58081 (10)	0.0295 (5)	
H11	0.1805	0.2676	0.5455	0.035*	
C12	0.4250 (5)	0.31536 (17)	0.62704 (11)	0.0291 (5)	
H12	0.3692	0.3851	0.6237	0.035*	

### Atomic displacement parameters (Å^2^)

**Table:**

	*U*^11^	*U*^22^	*U*^33^	*U*^12^	*U*^13^	*U*^23^
Br1	0.06219 (16)	0.03517 (14)	0.03072 (13)	0.00004 (16)	−0.01668 (12)	−0.00718 (8)
O1	0.0678 (12)	0.0294 (9)	0.0292 (9)	0.0169 (9)	−0.0064 (8)	0.0009 (7)
O2	0.0671 (12)	0.0285 (9)	0.0374 (10)	−0.0143 (9)	−0.0088 (9)	−0.0034 (7)
N1	0.0321 (10)	0.0195 (8)	0.0199 (8)	0.0021 (7)	0.0005 (7)	−0.0026 (6)
N2	0.0391 (9)	0.0173 (7)	0.0262 (8)	−0.0044 (10)	0.0027 (9)	−0.0003 (6)
C1	0.0400 (12)	0.0244 (11)	0.0261 (12)	−0.0031 (10)	−0.0012 (10)	−0.0022 (9)
C2	0.0597 (13)	0.0332 (11)	0.0235 (10)	0.0038 (16)	0.0059 (14)	0.0014 (8)
C3	0.0411 (13)	0.0314 (12)	0.0241 (11)	0.0036 (9)	0.0040 (9)	−0.0054 (9)
C4	0.0293 (11)	0.0230 (10)	0.0236 (10)	0.0028 (8)	0.0022 (8)	−0.0028 (8)
C5	0.0268 (11)	0.0250 (10)	0.0229 (10)	0.0004 (9)	0.0013 (8)	−0.0031 (9)
C6	0.0230 (11)	0.0221 (9)	0.0190 (9)	0.0013 (8)	0.0077 (8)	0.0005 (7)
C7	0.0288 (10)	0.0202 (10)	0.0231 (10)	−0.0035 (8)	0.0048 (9)	0.0011 (8)
C8	0.0316 (12)	0.0195 (9)	0.0318 (11)	0.0024 (10)	0.0010 (9)	−0.0039 (8)
C9	0.0273 (10)	0.0202 (10)	0.0226 (10)	−0.0005 (8)	0.0028 (9)	−0.0010 (8)
C10	0.0313 (10)	0.0271 (11)	0.0218 (11)	−0.0033 (9)	0.0014 (9)	−0.0043 (9)
C11	0.0359 (13)	0.0320 (11)	0.0205 (10)	0.0047 (10)	−0.0015 (9)	0.0051 (8)
C12	0.0383 (12)	0.0219 (11)	0.0271 (12)	0.0035 (9)	0.0050 (10)	0.0029 (9)

### Geometric parameters (Å, °)

**Table:**

Br1—C10	1.896 (2)	C3—H3B	0.9900
O1—C1	1.233 (3)	C4—C8	1.527 (3)
O2—C8	1.221 (2)	C4—H4	1.0000
N1—C1	1.339 (3)	C5—C6	1.500 (3)
N1—C4	1.465 (2)	C5—H5A	0.9900
N1—C5	1.465 (3)	C5—H5B	0.9900
N2—C8	1.356 (2)	C6—C9	1.391 (3)
N2—C7	1.422 (3)	C6—C7	1.406 (3)
N2—H2	0.8800	C7—C12	1.392 (3)
C1—C2	1.517 (3)	C9—C10	1.383 (3)
C2—C3	1.524 (3)	C9—H9	0.9500
C2—H2A	0.9900	C10—C11	1.379 (3)
C2—H2B	0.9900	C11—C12	1.386 (3)
C3—C4	1.533 (3)	C11—H11	0.9500
C3—H3A	0.9900	C12—H12	0.9500
			
C1—N1—C4	114.51 (16)	N1—C5—C6	113.00 (16)
C1—N1—C5	124.01 (17)	N1—C5—H5A	109.0
C4—N1—C5	121.36 (15)	C6—C5—H5A	109.0
C8—N2—C7	126.49 (16)	N1—C5—H5B	109.0
C8—N2—H2	116.8	C6—C5—H5B	109.0
C7—N2—H2	116.8	H5A—C5—H5B	107.8
O1—C1—N1	125.2 (2)	C9—C6—C7	118.99 (18)
O1—C1—C2	126.5 (2)	C9—C6—C5	119.95 (17)
N1—C1—C2	108.19 (18)	C7—C6—C5	120.95 (18)
C1—C2—C3	104.43 (17)	C12—C7—C6	119.90 (19)
C1—C2—H2A	110.9	C12—C7—N2	119.02 (18)
C3—C2—H2A	110.9	C6—C7—N2	120.98 (18)
C1—C2—H2B	110.9	O2—C8—N2	122.50 (19)
C3—C2—H2B	110.9	O2—C8—C4	121.76 (18)
H2A—C2—H2B	108.9	N2—C8—C4	115.73 (17)
C2—C3—C4	105.02 (17)	C10—C9—C6	119.74 (19)
C2—C3—H3A	110.7	C10—C9—H9	120.1
C4—C3—H3A	110.7	C6—C9—H9	120.1
C2—C3—H3B	110.7	C11—C10—C9	121.97 (19)
C4—C3—H3B	110.7	C11—C10—Br1	118.79 (15)
H3A—C3—H3B	108.8	C9—C10—Br1	119.20 (16)
N1—C4—C8	109.27 (16)	C10—C11—C12	118.56 (19)
N1—C4—C3	103.52 (16)	C10—C11—H11	120.7
C8—C4—C3	114.27 (17)	C12—C11—H11	120.7
N1—C4—H4	109.9	C11—C12—C7	120.8 (2)
C8—C4—H4	109.9	C11—C12—H12	119.6
C3—C4—H4	109.9	C7—C12—H12	119.6

### Hydrogen-bond geometry (Å, °)

**Table:**

*D*—H···*A*	*D*—H	H···*A*	*D*···*A*	*D*—H···*A*
N2—H2···O1^i^	0.88	2.00	2.864 (2)	169.
C5—H5A···O2^ii^	0.99	2.38	3.328 (3)	160.

Symmetry codes: (i) −*x*+2, *y*+1/2, −*z*+3/2; (ii) −*x*+2, *y*−1/2, −*z*+3/2.
